# Supplementary material for: A Multi-mRNA Host-Response Molecular Blood Test for the Diagnosis and Prognosis of Acute Infections and Sepsis: Proceedings from a Clinical Advisory Panel
Source: J Pers Med. 2020 Dec 7;10(4):266. doi: 10.3390/jpm10040266 (PMC7762405; doi:10.3390/jpm10040266)
Supplement: Supplementary file 1 [file jpm-10-00266-s001.pdf]

Supplementary Figures and Tables

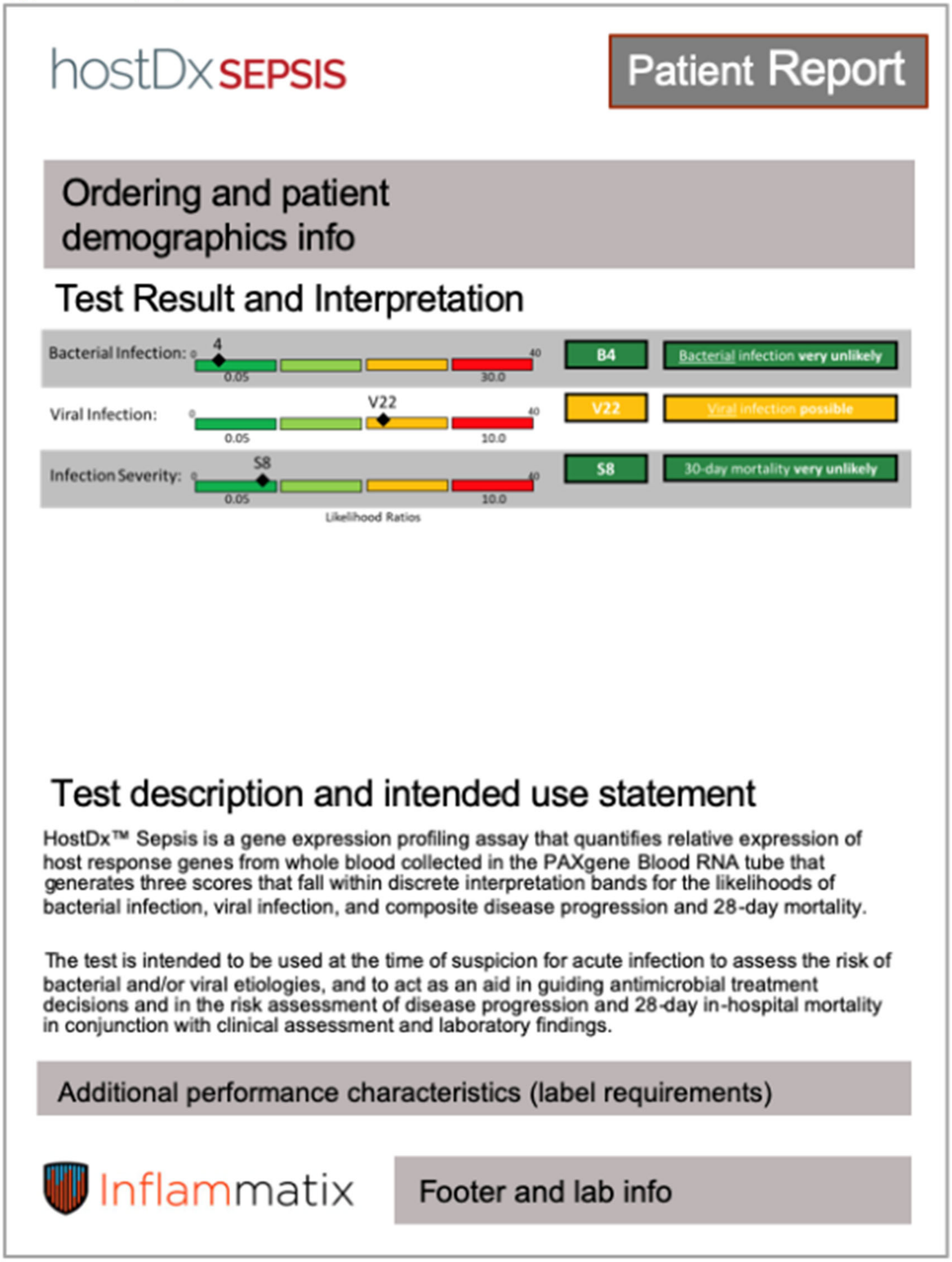

Suppl. Figure 1: Proposed patient report for InSep as presented to the advisory board. Horizontal representation of test result and interpretation. Adoption of a new test in the emergency room setting can only be successful if the required performance criteria are met (see below) and if the result readout (patient report) is easy to understand and actionable. The advisors agreed that presenting

the test results including the interpretation would be suitable as a patient report. Advisors appreciated the concept of having three distinct results (scores) presented. While it may complicate decision making compared to the current standard of care, it also yields novel opportunity such as a low bacterial/high viral score which in current diagnostic tests cannot be achieved. To make the test results immediately available to physicians in the EHR, simplified reports such as a combination of numbers and letters would be required; the report could however be visualized as a pdf in an EHR with uncertain level of usage by providers in a busy ER setting. To ensure that providers actually act upon the results presented the report must be compatible with EHR inclusion. The proposed reports were considered optically pleasing but could easily be misinterpreted since providers are used to looking at the color scales but not the scores. Therefore, providing results in words (e.g., likely, unlikely, indeterminate) would be a preferred simplification. Advisors also pointed towards the need for education on test performance and actionable results in addition to simplicity to ensure appropriate actions. Advisors also discussed the need to have sensitivity and specificity shown in the patient report. While having this information available is important, it will most likely not be used in the ED.

Supplementary Table S1: Modelled results for InSep results at a fixed prevalence of 50% with varying likelihood ratios of (A) 10, (B) 7.5, and (C) 5 for the very likely (rule-in) interpretation band.

(A)

Prevalence = 50%, LR low = 0.05, LR high = 10								
	**Neg**	**Pos**	**LR**	**% in band**	**NPV**	**PPV**	**Sens**	**Spec**
**Very likely**	28	281	**10.0**	**31%**	**68%**	**91%**	**56%**	**94%**
**Possible**	73	151	**2.07**	**22%**	**55%**	**68%**	**30%**	**86%**
**Unlikely**	218	59	0.27	28%	79%	61%	88%	44%
**Very unlikely**	181	9	0.05	19%	95%	61%	98%	36%

(**B)**

Prevalence = 50%, LR low = 0.05, LR high = 7.5								
	**Neg**	**Pos**	**LR**	**% in band**	**NPV**	**PPV**	**Sens**	**Spec**
**Very likely**	47	351	**7.49**	**40%**	**75%**	**88%**	**70%**	**91%**
**Possible**	68	91	**1.33**	**16%**	**51%**	**57%**	**18%**	**86%**
**Unlikely**	204	49	0.24	25%	81%	60%	90%	41%
**Very unlikely**	181	9	0.05	19%	95%	61%	98%	36%

(C)

Prevalence = 50%, LR low = 0.05, LR high = 5								
	**Neg**	**Pos**	**LR**	**% in band**	**NPV**	**PPV**	**Sens**	**Spec**
**Very likely**	83	415	**5.0**	**50%**	**83%**	**83%**	**83%**	**83%**
**Possible**	59	41	**0.7**	**10%**	**59%**	**51%**	**92%**	**12%**
**Unlikely**	177	35	0.19	21%	84%	59%	93%	35%
**Very unlikely**	181	9	0.05	19%	95%	61%	98%	36%

Supplementary Table S2: Modelled InSep results at a fixed prevalence of 50% with varying likelihood ratios of (A) 0.05, (B) 0.075, (C) 0.1, and (D) 0.15 for the very unlikely (rule-out) interpretation band.

(A)

**Prevalence = 50%, LR low = 0.05, LR high = 5**								
	**Neg**	**Pos**	**LR**	**% in band**	**NPV**	**PPV**	**Sens**	**Spec**
**Very likely**	83	415	**5.0**	**50%**	**83%**	**83%**	**83%**	**83%**
**Possible**	59	41	**0.70**	**10%**	**59%**	**51%**	**92%**	**12%**
**Unlikely**	177	35	0.19	21%	84%	59%	93%	35%
**Very unlikely**	181	9	0.05	19%	95%	61%	98%	36%

(**B**)

**Prevalence = 50%, LR low = 0.05, LR high = 5**								
	**Neg**	**Pos**	**LR**	**% in band**	**NPV**	**PPV**	**Sens**	**Spec**
**Very likely**	83	415	**5.0**	**50%**	**83%**	**83%**	**83%**	**83%**
**Possible**	59	41	**0.70**	**10%**	**59%**	**51%**	**92%**	**12%**
**Unlikely**	177	35	0.19	21%	84%	59%	93%	35%
**Very unlikely**	181	9	0.05	19%	95%	61%	98%	36%

(C)

Prevalence = 20%, **LR low = 0.1**, LR high = 10								
	**Neg**	**Pos**	**LR**	**% in band**	**NPV**	**PPV**	**Sens**	**Spec**
**Very likely**	45	112	10.0	16%	90%	71%	56%	94%
**Possible**	115	60	2.08	18%	83%	34%	30%	86%
**Unlikely**	115	14	**0.49**	**13%**	**89%**	**21%**	**93%**	**14%**
**Very unlikely**	524	13	**0.1**	**54%**	**98%**	**40%**	**93%**	**66%**

(D)

**Prevalence = 20%, LR low = 0.15, LR high = 10**								
	**Neg**	**Pos**	**LR**	**% in band**	**NPV**	**PPV**	**Sens**	**Spec**
**Very likely**	45	112	10.0	16%	90%	71%	56%	94%
**Possible**	70	48	2.73	12%	83%	41%	24%	91%
**Unlikely**	70	17	**0.95**	**9%**	**80%**	**19%**	**8%**	**91%**
**Very unlikely**	616	23	**0.15**	**64%**	**96%**	**49%**	**89%**	**77%**

Supplementary Table 3: Modelled InSep results at a fixed prevalence of 20% with varying likelihood ratios of (A) 10, (B) 7.5, and (C) 5 for the very likely (rule-in) interpretation band.
(A)

**Prevalence = 20%, LR low = 0.05, LR high = 10**								
	**Neg**	**Pos**	**LR**	**% in band**	**NPV**	**PPV**	**Sens**	**Spec**
**Very likely**	45	112	**10.0**	**16%**	**90%**	**71%**	**56%**	**94%**
**Possible**	116	60	**2.07**	**18%**	**83%**	**34%**	**30%**	**86%**
**Unlikely**	349	24	0.27	37%	94%	28%	88%	44%
**Very unlikely**	290	4	0.05	29%	99%	28%	98%	36%

(B)

Prevalence = 20%, LR low = 0.05, LR high = 7.5								
	**Neg**	**Pos**	**LR**	**% in band**	**NPV**	**PPV**	**Sens**	**Spec**
**Very likely**	75	141	**7.49**	**22%**	**92%**	**65%**	**70%**	**91%**
**Possible**	109	36	**1.33**	**15%**	**81%**	**25%**	**18%**	**86%**
**Unlikely**	326	20	0.24	35%	94%	28%	90%	41%
**Very unlikely**	290	4	0.05	29%	99%	28%	98%	36%

(C)

Prevalence = 20%, LR low = 0.05, LR high = 5								
	**Neg**	**Pos**	**LR**	**% in band**	**NPV**	**PPV**	**Sens**	**Spec**
**Very likely**	133	166	**5.0**	**30%**	**95%**	**56%**	**83%**	**83%**
**Possible**	94	17	**0.7**	**11%**	**85%**	**21%**	**92%**	**12%**
**Unlikely**	283	14	0.19	30%	95%	27%	93%	35%
**Very unlikely**	290	4	0.05	29%	99%	28%	98%	36%
